# Susceptibility and Transmission Dynamics of Peste des Petits Ruminants Virus in Domestic and Wild Ruminants: Experimental Insights from Kazakhstan

**DOI:** 10.3390/v17091231

**Published:** 2025-09-09

**Authors:** Lespek Kutumbetov, Balzhan Myrzakhmetova, Aiganym Tussipova, Gulzhan Zhapparova, Karina Bissenbayeva, Talshyngul Tlenchiyeva, Sergazy Nurabayev, Aslan Kerimbayev, Mukhit Orynbayev, Edil Makhashov, Kuandyk Zhugunissov

**Affiliations:** 1 Research Institute for Biological Safety Problems, Gvardeiskiy 080409, Kazakhstan; lespek.k@gmail.com (L.K.);; 2 Department of Veterinary Science, Kazakh National Agrarian Research University, Almaty 050002, Kazakhstan

**Keywords:** peste des petits ruminants (PPR), *Morbillivirus*, Cameroon goats, saigas, sheep, calves, virus transmission, susceptibility, experimental infection

## Abstract

Peste des petits ruminants (PPR) is an extremely contagious viral disease that significantly affects the health of small ruminants and the economies of livestock, particularly in areas adjacent to endemic regions. This study focused on assessing the vulnerability of different domestic and wild animal species in Kazakhstan, which includes local sheep and goats, African Cameroon goats, saigas, calves, and ground squirrels, to infection by the PPR virus (PPRV). Experimental infections used a virulent strain of PPRV (Nigeria 76/1), with animals being monitored over a period of 21–28 days to evaluate clinical signs, pathological lesions, and viral dissemination. The manifestation of disease differed across species, breed, and age. In Cameroon, goats and saigas displayed severe illness with a mortality rate of 100% and elevated virus levels in key organs, whereas local sheep and goats presented age-related subacute, abortive, and latent manifestations. Calves exhibited mild, subclinical infections, while ground squirrels showed no susceptibility. Viral shedding was observed in the secretions of infected animals, with transmission occurring through airborne and alimentary pathways. No virus carriage was detected in the animals that had recovered. The investigation emphasizes the notable variations in PPRV pathogenesis and transmission risk among different species, highlighting the necessity for focused surveillance and control strategies to avert incursions in PPR-free areas like Kazakhstan.

## 1. Introduction

Every nation faces the potential threat of foreign pathogens emerging during a time marked by the growth of global trade, tourism, and cultural interactions. It is crucial to maintain the vigilant protection of national territory against such threats, complemented by strong veterinary safeguards. Peste des petits ruminants (PPR) is recognized as a particularly significant threat among the various issues mentioned [1,2].

PPR is an infectious viral disease that significantly impacts both domestic and wild small ruminants. The condition is attributed to a *morbillivirus* belonging to the *Paramyxoviridae* family and is classified by the World Organisation for Animal Health (WOAH) as a notifiable disease because of its significant epizootological and economic implications [3]. Clinically, PPR presents with an acute or subacute onset of fever, ulcerative stomatitis, mucopurulent rhinitis, conjunctivitis, hemorrhagic gastroenteritis, lymphoid damage, and pneumonia. The morbidity can reach as high as 100%, with mortality rates soaring up to 90% in naive populations [4,5,6,7].

Initially limited to Africa [8], PPR has expanded its reach to various regions, including parts of Europe such as Turkey; Asia, with countries like China, Mongolia, and Thailand; and North Africa, encompassing Tunisia, Libya, and Algeria [9,10,11,12,13,14,15]. The socio-economic connections in these regions with Kazakhstan are robust, heightening the potential for PPRV introduction through avenues such as trade, tourism, or cross-border transhumance.

From January 2019 to October 2021, there were 164 officially reported outbreaks and 13 endemic cases of PPR, with incidents occurring in Burkina Faso, China, Kenya, Libya, Mongolia, Pakistan, Thailand, and Turkey [16,17,18,19,20]. Recent outbreaks in western Turkey and China [21,22] have led to mortality rates nearing 90%, highlighting the significant threat posed by the virus to Kazakhstan.

In light of this context, it is essential to assess the susceptibility of specific species, the dynamics of transmission, and the clinical and epidemiological features of PPRV in Kazakhstan. This information is essential for developing efficient surveillance, prevention, control, and potential eradication strategies customized to regional risk profiles. Creating robust, evidence-based defenses will strengthen veterinary health and protect small-ruminant industries from possible PPR incursions.

Prior to 2014, PPR had not been officially recorded in Kazakhstan, and the disease was considered exotic to the area. In 2014, reports indicated the presence of epizootic foci in the Zhambyl, Kyzylorda, and Almaty regions, where affected animals displayed clinical signs characteristic of PPR [23]. Nevertheless, the instances were not officially documented in veterinary records, and Kazakhstan maintains a PPR-free status as per the World Organization for Animal Health (WOAH).

While preventive vaccination initiatives using the live attenuated PPR vaccine from ARRIAH (Russian Federation) [24] are underway in the southern regions of the country, there is a lack of systematic epizootiological and serological surveillance. The absence of formal oversight prompts apprehensions regarding the possible unnoticed spread of the virus, especially in subclinical or mild instances that might escape detection.

Between 2022 and 2024, Kazakhstan did not report any confirmed outbreaks of PPR. In December 2022, a simulation exercise was conducted in the Zhambyl Region aimed at evaluating and enhancing veterinary preparedness for possible incursions. Even with the lack of documented cases within the country, PPR continues to pose a significant transboundary risk. From 2024 to 2025, there were more than 1280 reported outbreaks worldwide, indicating the ongoing epidemiological pressure of the virus [25].

This investigation aimed to tackle these issues by experimentally assessing the vulnerability of specific domestic animals (local sheep, local goats, calves) and wild species (saigas, ground squirrels), in addition to African Cameroon goats, to the PPR virus. The study seeks to define the clinical progression, pathological features, virus distribution, and potential for transmission of PPRV in these animals within a controlled environment. The study offers crucial insights into the disease’s epizootology by investigating variability in infection outcomes related to species, breed, and age. This includes identifying potential sources of infection and patterns of virus shedding. The results aim to enhance evidence-driven monitoring, risk evaluation, and response tactics within Kazakhstan’s veterinary framework while also contributing to wider regional and international initiatives focused on the containment and elimination of PPR.

## 2. Materials and Methods

### 2.1. Ethical Approval

The study was carried out in accordance with the principles outlined in the Declaration of Helsinki and received approval from the Institutional Review Board and Ethics Committee of the Research Institute for Biological Safety Problems, which is part of the Science Committee of the Ministry of Higher Education and Science of the Republic of Kazakhstan (Permit Number 1010/22).

### 2.2. Virus

The Nigeria 76/1 strain of PPRV was used for the purpose of animal infection. This strain was sourced from the Microorganism Collection Laboratory of the RIBSP.

### 2.3. Experimental Animals

The study included a total of 185 animals, comprising Kazakh merino sheep, local coarse-wool goats, Cameroon goats, saigas, Alatau calves, and yellow ground squirrels. Animals were grouped according to species and age, with the number of individuals per group varying depending on the experimental design. Detailed information on group composition is provided in Table 1. Animals were maintained in groups specific to their species under ABSL-3 conditions, with unrestricted access to food and water. All animals were subjected to a 7-day acclimatization period before the experimental infection started.

### 2.4. Cell Lines and Primary Cultures

The cell cultures used for virus isolation, titration, and neutralization assays included primary lamb kidney (LK) cells, primary goat kidney (GK) cells, primary saiga kidney (SK) cells, primary Siberian ibex kidney (SIBK) cells, the continuous sheep embryo kidney (SEK) cell line, and the Vero cell line derived from African green monkey kidney. All cell cultures were supplied by the Cell Biotechnology Laboratory of the RIBSP. Selection of each cell type was conducted with careful consideration of species specificity and susceptibility to morbilliviruses.

### 2.5. Nutrient Media

The cultivation of primary cell cultures (LK, GK, SK, and SIBK) was conducted using Dulbecco’s Modified Eagle Medium (DMEM; Sigma-Aldrich, St. Louis, MO, USA) supplemented with 5% fetal bovine serum (FBS; Gibco, Thermo Fisher Scientific, Waltham, MA, USA), penicillin (100 IU/mL), and streptomycin (100 µg/mL). Continuous cell lines, specifically Vero and SEK, were also cultured in DMEM with the addition of 10% FBS and the same antibiotic supplementation. All cell cultures were maintained under sterile conditions and incubated at 37 °C in a humidified environment with 5% CO_2_.

### 2.6. Animal Infection Methods

Experimental animals were subjected to infection through four distinct routes: subcutaneous, oral (alimentary), aerogenous, and contact exposure. However, the results presented in this manuscript are based exclusively on the subcutaneous infection model, as this route allowed for standardized delivery of the virus and reproducibility across all experimental groups. In the case of subcutaneous infection, subjects were administered 5.0 mL of virus-containing material, which comprised EDTA-treated blood, a 10% tissue suspension, or culture-grown virus exhibiting a titer between 3.0 and 5.0 log_10_ TCID_50_/cm^3^. Injections were delivered in the lower third of the neck, inner thigh, or shoulder area.

To induce oral infection, subjects were provided with feed that had been combined with a virus suspension (4.75 log_10_ TCID_50_/cm^3^) at a concentration of 1 mL of virus per 100 g of feed or remnants from infected subjects. In the context of aerogenous exposure, both infected and naïve animals were maintained in the same room under ABSL-3 conditions, yet they were kept in distinct enclosures to prevent any direct contact, as well as the sharing of food or water. To investigate contact transmission, healthy animals were placed in the same enclosure as infected animals for co-housing. Clinical observations were conducted twice each day. Rectal temperatures were recorded each morning and evening during the entire observation period.

### 2.7. Virus Isolation

#### 2.7.1. In Vivo Virus Isolation

For in vivo virus isolation, blood and tissue samples were used to assess viral infectivity via animal bioassay. Whole blood samples were collected from the jugular vein of experimentally infected animals into EDTA-treated vacutainer tubes. Tissue specimens—including lymph nodes, spleen, liver, kidneys, lungs, mucosal scrapings from the nose, mouth, abomasum, intestines, urinary bladder, and fetuses from aborted animals—were aseptically collected and homogenized in 10% (*w*/*v*) suspensions using Hanks’ balanced salt solution after a single freeze–thaw cycle. These homogenates were used to infect susceptible animals under controlled conditions for confirmatory pathogenicity testing. The appearance of clinical signs, viral antigen presence, and subsequent seroconversion were used to evaluate successful virus replication.

#### 2.7.2. In Vitro Virus Isolation

For in vitro virus isolation, the same sample types (whole blood, tissue homogenates, nasal and ocular discharges, saliva, and feces) were also processed for virus culture in cell monolayers. Secretions and excretions were collected using sterile swabs, placed in 4 mL of Hanks’ solution, and subjected to freeze–thaw cycles. The swabs were wrung out and discarded, and the eluates were centrifuged at 3000 rpm for 30 min. Supernatants (1.0 mL) were used to inoculate cell cultures.

All inoculated cell cultures were maintained at 37 °C in a 5% CO_2_ incubator. After a 1-h adsorption period, the inoculum was removed, cells were washed 5–6 times with Hanks’ solution, and fresh maintenance medium (DMEM with 2% FBS and antibiotics) was added.

Cultures were observed daily for cytopathic effect (CPE). If no CPE developed after 7–10 days, up to three blind passages were performed using 1.0 mL of supernatant. Viral identity was confirmed by CPE pattern, virus neutralization test, and PCR using the Bio-T kit^®^ Peste des Petits Ruminants (Biosellal, Chemin des Peupliers, Dardilly, France).

### 2.8. Virus Titration

In cell cultures, virus titration was conducted to ascertain the infectious titer of the material containing the virus. Tenfold serial dilutions of the viral samples were meticulously prepared in Hanks’ balanced salt solution. Next, 1.0 mL of each dilution was introduced into four wells of a culture plate that had been pre-seeded with confluent cell monolayers, following the removal of the growth medium.

For the negative control, 100 μL of Hanks’ solution devoid of virus was introduced to the control wells that housed uninfected cell monolayers. Following a one-hour period of virus adsorption at 37 °C, the inoculum was discarded, and the wells underwent rinsing. The cells were subsequently overlaid with maintenance medium, followed by incubation of the plates at 37 °C in a humidified 5% CO_2_ environment.

The maintenance medium underwent replacement every 2 to 3 days during the cultivation period. Daily observations were conducted on the plates to assess the cytopathic effect (CPE). The conclusive findings were documented on the fourteenth day. The virus titer was determined through the Reed and Muench method and presented as log_10_ TCID_50_/cm^3^.

### 2.9. Virus Neutralization Assay

The virus neutralization assay was conducted in LK, SK, SEK, and Vero cell cultures to identify virus isolates and detect specific virus-neutralizing antibodies in the sera of infected animals. Blood serum samples underwent inactivation at 56 °C in a water bath for a duration of 30 min before utilization.

Serial dilutions of each test serum were meticulously prepared in Hanks’ balanced salt solution, following a twofold approach. Equal volumes of diluted serum and viral suspension (containing 100–1000 TCID_50_/cm^3^) were combined and incubated at 37 °C for 1 h in a thermostat.

Following the incubation period, the mixtures were introduced into wells populated with confluent monolayers of LK, SK, SEK, or Vero cells. The cultures were maintained at 37 °C in a humidified atmosphere containing 5% CO_2_ for a duration of 14 days. The nutrient medium underwent replacement at intervals of 2 to 3 days.

The neutralizing antibody titer was established as the maximum serum dilution that entirely inhibited the occurrence of cytopathic effect (CPE) in a minimum of 50% of the inoculated wells.

CPE was deemed specific when it appeared in cultures inoculated with non-immune serum, while it was not present in cultures inoculated with immune serum.

### 2.10. Statistical Analysis

The analysis of data was conducted using Microsoft Excel 365. Virus titers (log_10_ TCID_50_/cm^3^) were determined employing the Reed–Muench method. The findings are displayed as mean ± standard deviation. Comparisons among groups (such as species, age, or clinical forms) were assessed using the Student’s *t*-test or one-way ANOVA, as applicable. A *p*-value < 0.05 was considered statistically significant.

## 3. Results

### 3.1. Susceptibility of Some Species of Domestic and Wild Animals to the PPRV

Researchers evaluated the susceptibility of local sheep to the PPRV across various age groups of local and Cameroonian goats, calves, saigas, and ground squirrels. Experimental infection was conducted through subcutaneous administration of a virulent 10% organ–tissue suspension of the Nigeria 76/1 strain (5.0 cm^3^ per animal) or a cultured virus (2nd to 5th passage in LK cell culture) at doses varying from 1000 to 100,000 TCID_50_. The duration of observation varied between 21 and 28 days.

The evaluation of susceptibility was conducted by analyzing the duration of the incubation period, clinical manifestations, disease progression, and outcomes. The specificity of PPRV infection was validated through reinfection with the homologous virus, virus isolation, PCR analysis of tissues from deceased animals, and serological assessment of paired sera from recovered animals using the virus neutralization assay.

The results indicated that local sheep and goats, Cameroon goats, calves, and saigas exhibit susceptibility to PPRV, with variations observed based on species, breed, and age. The susceptibility observed in local sheep and goats exhibited a significant dependence on age.

In Cameroon goats, the infection resulted in clear clinical disease in all goats and saigas. The disease manifested in a severe manner, exhibiting pronounced clinical signs, and resulted in a consistent fatal outcome.

In local sheep and goats, along with calves, the disease did not consistently manifest with observable clinical signs. The greatest percentage of clinically affected animals was observed in lambs and goats that were up to 3 months old. The majority of the infected animals in this group exhibited signs that are characteristic of PPR. In older young sheep (6–12 months), the percentage of clinically ill individuals varied between 68.8% and 83.9%; for goats of the same age, this figure was 85.7%.

The incidence of clinical disease in adult animals was observed to be lower, with rates of 31.8% in sheep, 60.0% in goats, and 50.0% in calves. The animals that remained in each group exhibited no apparent signs of infection throughout the entire observation period.

An adverse disease outcome was noted solely in young goats. The mortality rate was observed at 33.3% in animals up to 3 months old, while it was 7.1% in goats aged 6 to 12 months. No fatalities were recorded among adult animals, including sheep, saigas, or calves.

Ground squirrels infected with PPRV showed no observable clinical signs. Attempts to isolate the virus from organs and tissues collected on days 5, 7, and 14 post-infection using LK, SEK, and SK cell cultures did not yield successful results. Serum samples collected on day 30 post-infection showed no detection of virus-neutralizing antibodies, suggesting that there was no productive infection or seroconversion.

### 3.2. Course and Clinical Signs of the Disease

The study focused on the susceptibility of the PPRV to local sheep and goats, Cameroonian goats, saigas, calves, and ground squirrels. Researchers determined through careful observation of infected animals and thorough assessments of disease onset, severity, and clinical sign manifestation that the infection exhibits four distinct clinical forms: acute, subacute, abortive, and latent (subclinical).

The progression of infection across various animal species and age categories, as well as the associated morbidity and mortality rates, is detailed in Table 1.

Table 1 illustrates that an acute course of PPR was noted in all infected Cameroon goats (100%) and in the majority of saigas (88.2%). In a study of young local goats aged 1–3 months, it was observed that 14.8% exhibited acute cases, whereas only 3.7% of lambs in the same age group developed acute symptoms.

The subacute form was predominantly observed in goats aged 1–3 months (51.9%) and in lambs of the same age (22.2%). Instances of sporadic subacute forms were observed in older sheep, reaching up to 8.1%, as well as in goats, with a prevalence of up to 14.3%, and in saigas, which showed a rate of 11.8%.

The abortive form was noted solely in local sheep, goats, and calves. The occurrence was noted in 62.5–75.7% of sheep, varying by age, and in 64.3% of goats within the 6–12 month age range. In calves, an abortive course was observed in 50% of the animals.

The latent (subclinical) form was predominantly noted in older age groups: 68.2% of sheep over 18 months, 40.0% of goats of the same age, and 50% of calves exhibited no apparent clinical signs.

No indications of illness were detected in ground squirrels, and no virus was isolated from their tissues or blood; virus-neutralizing antibodies were not present in sera collected on day 30 after infection.

Table 2 provides a summary of the characteristics of clinical signs linked to each disease form.

The incubation period in the acute form spanned 2 to 3 days. The onset of the disease was characterized by a significant increase in body temperature, reaching levels between 41.0 and 41.8 °C, which continued throughout the duration of the illness. On days 2 to 3 of fever, the animals displayed signs of depression, fine muscle tremors, and hyperemia in the mucous membranes of the mouth, nose, and eyes. Necrotic lesions manifested on the mucous membranes of the lips, cheeks, gums, palate, pharynx, and tongue, resulting in shallow erosions characterized by irregular borders.

Salivation and a decrease in appetite were frequently observed. The conjunctiva exhibited edema, accompanied by vascular injection and crust formation resulting from purulent exudate. The nasal discharge has transitioned from a mucous consistency to a mucopurulent one. Certain animals exhibited a cough, with respiration becoming shallow and rapid, eventually leading to labored breathing accompanied by groaning in more severe instances.

The onset of stomatitis in local and Cameroonian goats was associated with gastrointestinal disturbances. Severe diarrhea manifested, characterized by liquid, dark green stools that included mucus and necrotic material. Instances of tenesmus and painful defecation were commonly noted. Animals assumed a position akin to that of a seated dog. In saigas, the onset of the disease frequently presented as constipation, which was subsequently followed by diarrhea. Not all of the animals that were affected exhibited symptoms of diarrhea.

In some pregnant animals, including two saigas and one Cameroon goat, spontaneous abortion occurred during the febrile stage of the infection. This was primarily due to high fever and systemic hyperthermia, which can disrupt fetal development and impair placental function. Additionally, the animals experienced significant nutritional and energy deficits caused by reduced feed intake, dehydration, and the increased metabolic demands associated with the inflammatory response. These combined stressors led to fetal death and subsequent abortion.

In specific saigas, the disease advanced more swiftly, resulting in mortality occurring between days 4 and 6. In certain instances, these animals exhibited solely fever and mucosal hyperemia, lacking any other significant clinical manifestations. The fever generally reached its highest point between 41.0 and 41.6 °C on the initial day, subsequently decreasing prior to the occurrence of sudden death.

The incubation period in the subacute form varied between 2 and 4 days. The body temperature increased to a range of 40.8–41.4 °C, although it was typically lower than observed in acute cases. In saigas, the fever presented as intermittent or brief episodes. Hyperthermia lasted for a duration of 5 to 8 days, with instances extending to 13 or 14 days.

On days 2 to 3, the subjects exhibited mild hyperemia in the mucous membranes of the oral and nasal cavities, conjunctiva, and areas of exposed skin. Localized necrotic lesions were observed, primarily affecting the soft palate and pharynx, with occasional involvement of the gums. The lesions manifested as superficial grayish-white coatings. Salivation was observed in certain animals.

Herpetic-like lesions and localized rashes were observed on the lips and skin of some lambs, typically lasting 2 to 3 days. These mucocutaneous changes were accompanied by conjunctivitis and rhinitis, though purulent nasal discharge was rare. As the febrile phase progressed, diarrhea developed in several animals, indicating gastrointestinal involvement. Goats and lambs affected by the subacute form of the disease exhibited marked weight loss, a dull and ruffled coat, and signs of mild depression. The weight loss resulted from decreased feed intake, elevated metabolic demand during fever, and systemic stress associated with viral infection.

In the majority of lambs and goats, gastrointestinal symptoms persisted for 2 to 4 days, subsequently leading to a swift recovery. In certain goats, diarrhea continued for a duration of 7 to 11 days, with affected animals either recovering gradually or succumbing to the condition.

The incubation period in the abortive form was observed to be between 4 and 7 days, with the duration of the disease ranging from 1 to 4 days. Affected animals exhibited mild hyperthermia, with temperatures ranging from 40.2 to 41.1 °C, alongside hyperemia of the mucous membranes. Several sheep exhibited herpetic lesions on their lips and rashes in hairless regions of their skin. No additional clinical signs were observed, and all animals made a complete recovery.

### 3.3. Pathological Changes

External examination of deceased animals showed significant emaciation and unkempt fur. Purulent exudate was noted in the medial canthi of the eyes, accompanied by dried accumulations. The area around the chin exhibited moisture attributed to salivation, while the nostrils showed signs of staining from mucopurulent discharge. The tail region and the fur on the hind limbs exhibited contamination from liquid feces. In certain saiga carcasses, the presence of bloody foam in the nasal and oral cavities was observed, suggesting significant pulmonary involvement and severe respiratory distress at the time of death.

The post-mortem examination of animals that succumbed to PPR infection demonstrated a comprehensive range of characteristic pathological lesions, particularly observed in goats from Cameroon and saigas. The observed animals exhibited significant and marked alterations at the organ level, characteristic of a virulent PPRV infection.

The pathological changes noted in the local children were relatively milder. Adult local sheep and goats that were euthanized due to illness exhibited infrequent gross pathological lesions, which, when present, were mild and not always distinctly identifiable, suggesting a possibly attenuated infection course or a recovery phase.

### 3.4. Pathomorphological Changes in Organs

#### 3.4.1. Digestive System

In Cameroon goats, goats and saigas that succumbed to PPR exhibited pronounced necrotic and inflammatory alterations in the digestive system. Erosions and ulcers of varying depth and size were observed in the oral cavity, affecting the gums, lips, soft palate, pharynx, and tongue. These lesions were frequently covered with necrotic debris and were accompanied by areas of hyperemia and edema. Local goats exhibited comparable, albeit milder, lesions—generally confined to the soft palate and pharyngeal region.

The esophagus and forestomachs, including the rumen, reticulum, and omasum, exhibited a typical appearance, with moderate quantities of feed present. In certain instances, there was a noted presence of mild hyperemia in the omasal folds.

The abomasal mucosa exhibited congestion and edema, particularly in the pyloric region, accompanied by small hemorrhages. In two saigas, there were deep ulcerations extending into the muscular layer.

The small intestine frequently exhibited emptiness or was filled with a watery chyme. The duodenal wall showed signs of thickening, edema, and increased blood flow, accompanied by localized petechiae and areas of necrosis. In the jejunum and ileum, there were frequent observations of mucosal injection and necrotic plaques, accompanied by hemorrhages and gray-white caseous deposits. A notable lesion appeared on the ileocecal valve as a red, sharply defined hyperemic rim. In numerous saigas and certain goats, the small intestine exhibited a widespread dark red coloration as a result of significant hemorrhages.

The large intestine housed unformed, frequently malodorous feces. The mucosal surfaces of the cecum and colon exhibited edema and were covered with caseous material. Notable necrotic regions, along with mucosal thinning and a range of hemorrhagic manifestations from petechiae to streaks, were prominently observed in the cecum. Conversely, the rectum typically remained unaffected.

The liver exhibited a dark brown hue and congestion, characterized by a smooth surface and indications of dystrophy in certain saigas. The gallbladder exhibited distension filled with dark green bile.

In adult sheep and goats that were euthanized during the disease, gastrointestinal lesions were restricted to mild mucosal hyperemia and edema observed in the duodenum and jejunum.

The findings indicate that severe and systemic involvement of the digestive tract was characteristic in saigas and Cameroon goats, whereas local sheep and goats, especially adults, exhibited milder or localized changes.

#### 3.4.2. Respiratory Organs

The nasal cavity, larynx, trachea, and bronchi displayed signs of catarrhal to catarrhal purulent inflammation. The mucosa exhibited hyperemia and was associated with mucous or mucopurulent exudate. In certain saigas and goats, the presence of bloody foam was observed, suggesting pulmonary edema.

The lungs exhibited a range of colors from pink to dark red, accompanied by regions of consolidation and infiltration. In the examined section, the impacted lobes exhibited a firm texture, were moist, and demonstrated an absence of sponginess. In severe instances, gray–red hepatization along with small purulent foci was noted. Fibrinous deposits were sometimes observed on the pleura. Respiratory lesions were evident in saigas and Cameroon goats, with pneumonia being a recurring observation. The involvement of local animals exhibited variability, typically presenting a milder degree.

#### 3.4.3. Lymphoid Organs

The prescapular, submandibular, retropharyngeal, and mesenteric lymph nodes exhibited enlargement, tension, and edema. In this section, the nodes exhibited a moist, smooth surface along with congested parenchyma. In certain instances, hemorrhages were noted, particularly within the mesenteric nodes.

The spleen exhibited occasional enlargement, presenting a dark red hue and a flaccid texture, characterized by soft pulp and indistinct margins. The observed changes indicate a robust immune response and systemic inflammation, especially in saigas and goats exhibiting severe disease symptoms. The lymphoid alterations observed in adult sheep and goats were either minimal or completely absent.

#### 3.4.4. Urinary Organs

The kidneys exhibited a dark red hue and a flaccid texture, characterized by smooth capsules that could be effortlessly detached. The delineation between the cortex and medulla was inadequate. The renal pelvis exhibited mild edema.

The urinary bladder exhibited contraction and held 5–20 mL of straw-colored urine. The mucosa exhibited hyperemia. Lesions in the urinary tract were found to be nonspecific and predominantly noted in animals experiencing prolonged or systemic disease.

#### 3.4.5. Heart and Vessels

The myocardium exhibited a pale and flaccid appearance. The cardiac chambers were filled with inadequately coagulated blood. Petechial and streaked hemorrhages were commonly observed on the endocardial and epicardial surfaces, especially in the regions of the left ventricle and atria.

Cardiac lesions served as indicators of widespread vascular impairment and advanced toxemia. These occurrences were observed more frequently in saigas and juvenile goats presenting with acute illness.

### 3.5. The Role of Infected Animals in the Epizootic Process

A study investigating PPR across different animal species revealed varying degrees of susceptibility to the disease. Nonetheless, the mere presence of susceptibility is not enough to ascertain the epidemiological importance of a species. To elucidate the role of infected animals in the epizootology of PPR, it is crucial to evaluate factors such as the presence and duration of viremia, viral localization in internal organs, the intensity and timing of viral shedding into the environment, the potential for virus carriage, and possible transmission routes.

The existing literature offers a brief overview of the involvement of specific African animal species in the transmission of PPR; however, there is a notable absence of data regarding animals raised in European and Asian regions. Consequently, this investigation sought to explore these epizootological facets using susceptible animal models within regulated experimental settings.

### 3.6. Viremia and Viral Localization in the Body

Daily blood samples were collected from infected animals over a period of 12–14 days post-infection to assess the presence and duration of viremia. This period aligned with the incubation phase, the progression of the illness, and the initial stages of recovery.

Table 3 summarizes the results of viremia detection across various species and age groups.

Table 3 indicates that significant viremia was predominantly observed in local goats within the age range of 1 to 3 months. The virus was predominantly identified in these animals during the period from days 4 to 9 following infection. Viral RNA was identified in one goat from day 4 through day 13. In lambs within the same age group, viremia was detected from day 3 or 4 until day 7, although not all infected lambs yielded positive test results. In older lambs and goats, the virus was identified infrequently, usually coinciding with peak hyperthermia, occurring between days 5 and 7 post-infection.

No virus was detected in the blood samples of sheep older than 12 months or in Cameroon goats during the entire observation period. In saigas, viremia was transient, lasting only 1 to 3 days.

To investigate the localization and accumulation of the virus in internal organs, post-mortem samples were obtained from animals that either succumbed or were euthanized during the clinical phase of the disease. Samples were collected from saigas and local goats aged 1 to 3 months during days 3 to 5 of the illness. Tissues were collected postmortem from goats in Cameroon after spontaneous death occurred. During the clinical phase of illness, specifically between the 3rd and 5th day, organ samples were collected from older sheep and goats. Detection of the virus was carried out through isolation in lamb kidney (LK) cell culture or by infecting naïve animals. The quantification of viral titers in organs was performed through cell culture titration, with identity verification achieved via PCR and virus neutralization tests using PPRV-specific serum.

The results regarding viral localization and accumulation in the organs of sheep, goats, and saigas are detailed in Table 4.

Table 4 illustrates that PPRV was primarily found in the organs associated with the digestive tract, respiratory system, and lymphoid tissues of the infected animals. The tissues of Cameroon goats exhibited the highest viral titers, with concentrations reaching 4.5–5.5 log_10_ TCID_50_/cm^3^ in the gastrointestinal mucosa, 4.24–5.0 log_10_ TCID_50_/cm^3^ in the nasal mucosa and lungs, 0.75–2.25 log_10_ TCID_50_/cm^3^ in the lymph nodes and spleen, and 0.5 log_10_ TCID_50_/cm^3^ in the kidneys.

Saigas that were euthanized or found deceased on days 3 to 5 of illness typically had lower viral titers. The presence of the virus was identified at levels ranging from 2.50 to 3.75 log_10_ TCID_50_/cm^3^ in the mucosal tissues of the digestive tract, nasal passages, and lungs, with concentrations reaching up to 4.5 to 4.75 log_10_ TCID_50_/cm^3^ in the lymph nodes and spleen. The virus was not successfully isolated from the liver or kidneys of saigas.

In local goats aged 1–3 months, the virus was detected at 0.75–1.5 log_10_ TCID_50_/cm^3^ in the lymph nodes, spleen, and intestinal mucosa of euthanized animals. In animals that succumbed to natural causes, the isolation of the virus was achievable solely from lymph nodes and intestinal tissues, and this was only after conducting 2–3 blind passages in LK cell culture. No viral presence was identified in the other organs of these subjects.

In adult sheep and goats, the isolation of the virus was infrequent and confined to specific lymph nodes, necessitating 2–3 passages in cell culture for successful detection. The findings suggest that viremia in local sheep, goats, and saigas aligns with the clinical stage of infection, whereas viremia was not present in Cameroon goats despite significant viral accumulation in their internal organs. The virus was predominantly found in the digestive, respiratory, and lymphoid organs, with the highest concentrations observed in Cameroon goats and saigas. Conversely, in local sheep and goats, the viral load was comparatively low, with the virus being most reliably isolated from the prescapular lymph nodes.

### 3.7. Virus Isolation and Carriage

The assessment of PPR virus (PPRV) in different secretions and excretions from infected animals was conducted to identify possible pathways for viral shedding and transmission. Samples were gathered from sheep, goats, and saigas exhibiting clinical manifestations of the disease, which included nasal and oral discharges (saliva and mucus), ocular secretions (tears), urine, and feces. Isolation of the virus was conducted using primary lamb kidney (LK) cell cultures. The findings are presented in Table 5.

In local breeds, the shedding of the virus was most evident in goat kids between the ages of 1 and 3 months. PPRV was identified in the ocular and nasal secretions of these animals as early as days 1–2 of the febrile period and in oral secretions and feces within 1–2 days following that. The length of time and the amount of virus excreted were linked to the clinical presentation and severity of the illness. In instances of acute and subacute PPR, the shedding of the virus continued for a duration of 5 to 6 days, with certain cases extending to 8 or 9 days. In animals exhibiting an abortive course, the duration of virus excretion was confined to a span of 2–3 days. Virus titers in secretions varied between 0.75 and 2.5 lg TCID_50_/cm^3^.

In older goats and sheep, the excretion of the virus was characterized by its intermittent nature, brevity, and occurrence at low titers. Detecting the virus in these groups necessitated the inoculation of whole samples or conducting 2–3 blind passages in cell culture. In goats aged 6 months, viral shedding was observed for a duration of 2 to 3 days through nasal, ocular, and fecal pathways. In goats exceeding 12 months of age, the virus was exclusively isolated from fecal matter. In sheep aged 6 months, the virus was identified in nasal discharge and saliva for a duration of 2 to 3 days, with less consistent detection in ocular discharge. In older sheep, PPRV was found solely in nasal secretions, with no presence in feces.

The virus in saigas was identified in nasal, ocular, and oral secretions starting 2–3 days following the onset of fever. Fecal shedding was observed at a later stage. During the progression of the disease, viral titers in secretions and feces showed a gradual increase, ultimately reaching peak levels of 4.0–4.25 lg TCID_50_/cm^3^ in the terminal stages. In the final days leading up to death, there was a slight decline in titers observed in individual animals.

Cameroon goats that were infected started to shed the virus by the second day of exhibiting fever, showing measurable levels in nasal, ocular, and oral secretions, in addition to feces. At the outset, titers were observed between 0.5 and 1.75 lg TCID_50_/cm^3^, experiencing a significant increase in the following days. Maximum titers were recorded in feces (up to 4.5), saliva (3.5–3.75), nasal discharge (3.5–4.75), and ocular discharge (4.0–5.25 lg TCID_50_/cm^3^). Elevated viral loads remained present until the demise of the subjects. In one individual, a decrease in body temperature (from 37.5 °C to 34 °C) three days before death was observed alongside a significant decline in virus titers in secretions, with some samples showing undetectable levels of the virus.

The results demonstrate that saigas and Cameroon goats are significant shedders of PPRV, releasing the virus in high concentrations during the progression of the disease. Local goat kids aged 1 to 3 months showed viral excretion, but this occurred at reduced titers and for shorter durations. Sheep and older goats exhibited restricted viral shedding, typically occurring between days 2 and 6 of illness and at low concentrations.

### 3.8. Assessment of Post-Recovery Virus Carriage

To assess the potential for post-recovery virus shedding, sheep and goats that had recovered from PPRV infection were monitored for 30 days. Blood, secretions, and excretions were collected regularly, and tissue samples were taken at the end of the period. All samples tested negative for viral presence in both cell culture and animal bioassays. Additionally, three goat kids inoculated with blood and tissue suspensions from recovered animals showed no clinical signs or antibody response. Four healthy animals co-housed with recovered ones for 30 days also remained clinically healthy and seronegative but became infected when later exposed to live virus. These findings indicate that recovered animals do not shed PPRV and are unlikely to contribute to transmission.

## 4. Discussion

PPR is now acknowledged as a distinct disease entity resulting from a morbillivirus belonging to the Paramyxoviridae family. Initially identified in Côte d’Ivoire in 1942 [26,27], it stayed limited to Africa until the late 1980s, resulting in significant losses in sheep and goat farming. In Nigeria, it has been estimated that annual economic losses reach approximately USD 1.5 million [28,29].

Since the 2000s, PPR has broadened its reach into Europe, Asia, and the Middle East. The initial documented case emerged in Georgia in 2016, while strains of PPRV that were closely associated with those from China were identified in Kazakhstan during the years 2014–2015 [30,31,32,33].

To examine susceptibility in domestic livestock and wildlife within Kazakhstan, we conducted experimental infections on sheep, goats (both local and Cameroon breeds), calves, saigas, and ground squirrels. The findings of our studies demonstrated variation in PPRV infection dynamics that is influenced by species, breed, and age.

Cameroon goats and saigas exhibited acute or subacute disease with a mortality rate of 100%, highlighting their significant susceptibility irrespective of age. This underscores their capacity to serve as enhancing hosts, especially in environments with multiple species or in zoological contexts.

Local sheep and goats demonstrated a distinct susceptibility that varied with age. Goat kids under 3 months often experienced acute or subacute illnesses, with mortality rates ranging from 7.1% to 33.3%. Goats and sheep older than 12 months generally underwent abortive infections, demonstrating full recovery without fatalities—this aligns with earlier observations.

Calves exhibited restricted susceptibility, frequently undergoing abortive infections without pronounced clinical symptoms, indicating a possibility of spillover yet minimal epidemiological relevance in natural settings.

Ground squirrels and other tested rodents demonstrated a complete lack of susceptibility to PPRV, with no signs of infection or serologically detectable exposure observed. This reinforces the idea that PPRV exhibits a high degree of host specificity [34,35].

The findings indicate that PPR shows a significant reliance on factors such as species, breed, and age-related susceptibility. This conclusion is consistent with the observations made by various authors who have investigated the disease in both endemic and epizootic regions [9,36,37]. The experimental findings broaden the recognized host range by including Kazakh merino sheep, coarse-wool goats, saigas, and calves as susceptible species, which points to the epidemiological importance of both domestic and wild ruminants in Central Asia [38,39,40].

The disease presented in both typical and atypical clinical forms, with the typical forms—including acute and subacute PPR—showing clinical signs that align with those noted in previous reports: sustained hyperthermia (41.2–41.8 °C), necrotic stomatitis, mucopurulent rhinitis and conjunctivitis, and diarrhea [41,42]. The subacute form exhibited milder clinical expression and a more prolonged disease course, generally linked to partial resistance in older or previously exposed animals.

The unusual presentations, including abortive and latent (subclinical) infections, were marked by mild hyperthermia (40.5–41.1 °C), transient mucosal hyperemia, occasional skin lesions, or a total lack of clinical signs. It is noteworthy that skin lesions, while not consistently observed, have been documented in milder cases of PPR and regarded as a sign of a relatively benign progression of the disease [43]. This evidence indicates that skin-related issues might signify reduced viral activity or adaptation by the host.

The findings from the pathological examination exhibited a strong correlation with the severity of the clinical condition. Lesions were consistently noted in the digestive tract, respiratory system, and lymphoid organs in both acute and subacute forms, exhibiting characteristic catarrhal hemorrhagic inflammation and ulcerative necrotic changes. Conversely, abortive and latent forms often exhibited an absence of macroscopic lesions, potentially complicating diagnosis in field conditions. The observed patterns align with earlier investigations detailing the pathogenesis of PPR and its tissue tropism in small ruminants [44,45].

Consequently, the clinical and pathological features of PPR identified in our investigation align with established manifestations of the disease and ought to be regarded as dependable indicators of infection in both conventional host species and non-conventional or local breeds, such as Kazakh merino sheep, coarse-wool goats, saigas, and calves. The findings enhance the overall comprehension of PPR’s epidemiology and have the potential to guide surveillance and diagnostic protocols in areas where mixed livestock and wildlife populations are present.

The notable variations identified between local and African-origin breeds in the clinical progression of PPR can be attributed to species- and breed-specific susceptibility, as well as the occurrence of secondary infections in endemic regions. Field investigations have often indicated that concurrent infections—such as *Pasteurella multocida*, *Klebsiella pneumoniae*, and *Mycoplasma* spp.—significantly worsen the severity of PPR, leading to elevated mortality rates due to bronchopneumonia and septicemia [46,47]. Under our controlled experimental conditions, similar to other studies, we found no evidence of secondary pathogens. This result underscores the possibility that coinfections may contribute to severity in natural outbreaks, despite their absence in laboratory settings.

Our virological investigations provided additional evidence for variations in host susceptibility and linked these findings to the pathologic changes observed. Our findings indicate that viremia occurred alongside febrile episodes, with the highest viral loads consistently observed in tissues exhibiting visible lesions, specifically in the mucosa of the digestive and respiratory tracts as well as in lymphoid organs. This distribution corresponds with the established tissue tropism of PPRV, which shows a preference for replication in lymphoid and epithelial tissues.

There was a strong correlation between virus titers and the severity of the disease:

- In acute instances with Cameroon goats and saigas, viral loads were observed at 4.75–5.5 log_10_ TCID_50_/cm^3^, suggesting systemic and high-level replication;
- In subacute infections, such as those affecting local goat kids and lambs, titers were recorded at lower levels—up to 1.5 log_10_ TCID_50_/cm^3^—indicating moderate pathogenicity;
- In abortive cases, the presence of low-level virus necessitated 2–3 blind passages for detection, highlighting limited replication and early containment by the host immune response.

Our findings confirm a strong link between disease severity, host species, and tissue-specific viral distribution. Notably, PPRV showed preferential replication in mucosal tissues of Cameroon goats, while in saigas, lymphoid organs were the primary sites of viral accumulation. These interspecies differences in viral tropism suggest that PPRV replication is influenced by host-specific immune or cellular factors, a pattern consistent with previous observations [36,48,49].

The observed viral distribution in local sheep and goats exhibited a pattern akin to that of saigas, albeit with generally lower titers, indicating a degree of partial resistance or diminished viral replication capability. A significant observation was the variation in viremia. In Cameroon goats, viremia was not detected during the febrile or clinical period, a finding that aligns with results from other studies involving highly susceptible African goat breeds [10,50].

Conversely, local sheep and goats consistently exhibited detectable viremia, irrespective of the severity of clinical signs. In saigas, viremia was observed, though it was brief, generally coinciding with the febrile stage. The observed differences can be linked to the unique immune responses associated with specific breeds and species, which affect the dynamics of systemic viral dissemination.

Collectively, these findings reinforce the idea that the progression of PPRV infection and the distribution pattern of the virus within tissues are significantly shaped by the host’s immune response and the vulnerability of specific target cell populations. The responses specific to the host may play a crucial role in deciding if the virus stays confined or spreads throughout the system. From a diagnostic perspective, the evidence indicates that the selection of tissue for virus detection must be customized according to the specific species involved. In Cameroon goats, respiratory and gastrointestinal tissues in goats are more likely to produce positive virological results, while lymphoid tissues may provide more insightful information in saigas.

This information is especially crucial for enhancing diagnostic sensitivity, particularly when employing methods such as RT-PCR or virus isolation. This also carries important implications for investigations into outbreaks and studies of pathogenesis, underscoring the necessity for tailored approaches specific to each species in the realms of disease surveillance and control. Furthermore, the noted variations in viremia highlight the constraints of depending exclusively on blood-based diagnostics in specific species, especially in instances where the virus is rapidly sequestered in tissues.

Extensive information regarding the epizootological dynamics of PPR—encompassing sources of infection, mechanisms of virus carriage, transmission pathways, and environmental resilience—continues to be scarce. Current data, primarily obtained from field observations, suggests that clinically ill animals act as the main reservoirs, with transmission taking place through close contact [12,51]. The role of wildlife in PPR epizootology is still not well understood, even though evidence from other diseases indicates that wild species can serve as reservoirs for pathogens that impact domestic animals [21,52,53].

The findings from our experimental investigations indicate that infected animals, both domestic (such as sheep, local goats, and Cameroon goats) and wild (like saigas), actively excrete PPRV during the febrile and clinical phases. Viral shedding was observed through nasal and ocular discharges, saliva, and feces, with the intensity of shedding directly linked to the severity of the disease and the susceptibility of the host. During acute infections, Cameroon goats and saigas exhibited virus titers reaching 4.75 log_10_ TCID_50_/cm^3^ and 4.25 log_10_, respectively, whereas local lambs and goat kids released lower titers, peaking at 2.5 log_10_. In milder subacute and abortive cases, the duration and level of viral excretion were both reduced.

Importantly, horizontal transmission to co-housed healthy animals was consistently noted, irrespective of species or age, validating both airborne and alimentary pathways of dissemination.

The determined contagiousness index—the probability of transmission in a co-housing scenario—varied from 0.66 to 1.00 when the donor was a highly susceptible species, like a Cameroon goat or saiga, and the recipient was an unexposed sheep, goat, or saiga. Conversely, the transmission from adult local sheep and goats yielded a significantly lower index (0.12–0.37), which likely indicates their diminished viral excretion. The findings emphasize the need to consider both the intensity of viral shedding and the susceptibility of the recipient as critical factors influencing transmission dynamics.

The results of our study demonstrate that direct contact and mucosal secretions play a key role in the transmission of PPR. The identification of saigas—a wild species—as both a source of virus shedding and a potential vector is especially important, providing new insight into the involvement of wildlife in the spread of this disease.

Infected animals are the established source of PPRV, and the transmission of the disease occurs through close interactions in mixed-species environments, particularly when high-shedding species are present. The insights gained are essential for guiding specific control strategies, including the isolation of infected animals, improved biosecurity measures in mixed-species herds, and vaccination initiatives aimed at high-risk species to halt the transmission of infection.

The data highlight that, following the introduction of PPR into a population, the disease can disseminate swiftly among highly susceptible species, saigas and Cameroon goats, which are often present in zoological collections within the country. Both species exhibited rapid disease progression and significant viral shedding, positioning them as effective amplifiers of the virus during outbreaks.

Conversely, local sheep and goats exhibited a more gradual disease transmission, frequently showing mild or subclinical symptoms. The infection observed in these animals often took an abortive or latent trajectory, leading to positive outcomes. Nonetheless, this seemingly benign nature presents an epizootiological concern: the unnoticed spread of the virus among flocks could remain hidden, particularly without regular clinical and serological monitoring. As a result, PPR can subtly disseminate among populations of seemingly healthy animals, facilitating broader transmission across flocks and regions.

A major challenge in managing PPR in enzootic countries is the presence of latent infections in adult sheep and goats, especially in specific breeds that do not exhibit clear symptoms of illness yet still seroconvert.

Asymptomatic carriers can serve as hidden sources of infection, particularly during environmental stressors like the rainy season or animal transport—situations recognized to provoke reactivation of subclinical infections and elevate virus excretion [3,45,51].

Our controlled experiments yielded no evidence of ongoing virus carriage in sheep and goats that have recovered from PPR. The absence of viral RNA in convalescent animals, as indicated by both virus isolation and PCR detection, implies that these post-recovery animals are unlikely to act as long-term reservoirs for PPRV transmission. This finding is consistent with multiple earlier studies that indicate PPRV does not create chronic infections, setting it apart from other morbilliviruses such as measles, which can occasionally persist in neural tissues [45].

Nonetheless, serological surveillance remains an essential method for detecting virus-neutralizing antibodies in previously exposed populations and pinpointing areas of silent viral circulation, especially in regions where the clinical burden is minimal or sporadic. This method is essential for guiding vaccination strategies and focused disease management, particularly in mixed herds or wildlife interfaces.

## 5. Conclusions

The results of this investigation demonstrate that PPRV shows notable pathogenicity that varies by species, breed, and age. Local sheep and goats exhibited different levels of susceptibility influenced by age, as younger animals showed more pronounced clinical signs and higher mortality rates, whereas adults typically presented with subclinical or abortive manifestations. In contrast, Cameroon goats and saigas exhibited significant susceptibility, characterized by swift disease advancement, pronounced clinical symptoms, and a mortality rate of 100%—identifying them as high-risk amplifiers of the virus in potential outbreak situations.

The detection of PPRV in secretions like nasal discharge, saliva, tears, and feces, along with its effective transmission among and between species in experimental settings, highlights the effectiveness of both airborne and alimentary transmission pathways. Nonetheless, the lack of virus detection in urine and the absence of carriage in recovered animals indicate a minimal risk of prolonged virus persistence following recovery.

The complete resistance of ground squirrels to PPRV infection highlights the virus’s host specificity, effectively excluding these rodents from the epizootic chain. The findings indicate that employing clinical signs, virus isolation, and serological testing is effective for diagnosis and monitoring purposes. Considering Kazakhstan’s geographic proximity to endemic nations, this study offers crucial data for early detection, species-specific risk evaluation, and the formulation of targeted biosecurity measures to avert future PPR incursions.

## Figures and Tables

**Table 1:** Susceptibility to the PPR virus of local Kazakh breeds of sheep and goats, Cameroon goats, calves, saigas, ground squirrels, and the course of the disease in them.

Species and Breed of Animals	Age of Animals (Month)	Number of Infected Animals (Head)	The Course of the Disease and the Number of Sick Animals				Fatality %
			Acute (Heads/%)	Sub-Acute (Heads/%)	Abortive (Heads/%)	Latent (Heads/%)	
Local sheep	1–3	27	1/3.7	6/22.2	17/63.0	3/11.1	-
	6	37	-	3/8.1	28/75.7	6/16.2	-
	12	16	-	1/6.3	10/62.5	5/31.2	-
	18 and older	22	-	-	7/31.8	15/68.2	-
Local goats	1–3	27	4/14.8	14/51.9	6/22.2	3/11.1	33.3
	6–12	14	1/7.1	2/14.3	9/64.3	2/14.3	7.1
	18 and older	5	-	-	3/60.0	2/40.0	-
Cameroon goats	4–24 and older	6	6/100	-	-	-	100
Saiga	6 and older	17	15/88.2	2/11.8	-	-	100
Calves	8–10	4	-	-	2/50	2/50	-
squirrels	young and adults	10	-	-	-	-	-

**Table 2:** The nature of clinical signs of PPR in various forms of the course.

Disease Form	Species	Baseline Temp (°C)	Incubation Period (Days)	Max Temperature (°C)	Stomatitis	Rhinitis	Conjunctivitis	Diarrhea	Skin Lesions	Duration (Days)	Mortality (%)	Recovery (%)
Acute	Cameroon goats	39.5 ± 0.3	2–3	41.2–41.3	++	++	++	++	−	7–10	100	−
	Saiga	39.4 ± 0.2	2–3	41.2–41.7	++	++	++	−	−	6–8	100	−
	Local goats	39.5 ± 0.3	2–3	41.4–41.8	++	++	++	++	−	8–11	100	−
	Local sheep	39.6 ± 0.4	2	41.6–41.8	++	++	++	+	−	10	−	100
Subacute	Saiga	39.3 ± 0.2	3–4	40.8–41.1	+	+	+	+	−	10–14	−	100
	Local goats	39.4 ± 0.3	3–4	41.0–41.2	+	+	+	++	−	5–8, 13	25	75
	Local sheep	39.2 ± 0.2	2–4	41.0–41.4	+	+	+	−	++	5–7	−	100
Abortive	Local goats	39.3 ± 0.3	4–6	40.5–41.1	−	−	−	−	−	2–4	−	100
	Local sheep	39.1 ± 0.2	4–7	40.5–41.0	−	−	−	−	+	1–3	−	100
	Calves	38.6 ± 0.3	4–5	40.2–40.4	−	−	−	−	−	1–2	−	100

Note: «++»—the clinical sign is clearly expressed; «+»—the clinical sign is weakly expressed; «−»—the clinical sign is absent.

**Table 3:** Results of the study of viremia in PPR in sheep, goats, and saigas.

Species and Breed of Animals	Age of Animals (Months)	Presence of the Virus in the Blood per Day After Infection													
		2	3	4	5	6	7	8	9	10	11	12	13	14	15
Saiga	6–24	0/3	1/3	2/3	0/3	1/2	0/1	-	-	-	-	-	-	-	-
Cameroon goats	4–24	0/3	0/3	0/3	0/3	0/3	0/3	0/3	0/3	0/2	0/1	-	-	-	-
Local coarse-wool goats	3	0/4	0/4	2/4	3/4	4/4	4/4	4/4	1/4	1/4	1/4	1/4	1/4	0/4	-
	6	0/4	0/4	0/4	3/4	4/4	3/4	0/4	0/4	0/4	0/4	0/4	0/4	0/4	-
	12	0/3	0/3	0/3	1/3	2/3	0/3	0/3	0/3	0/3	0/3	0/3	0/3	0/3	-
Local merino sheep	3	0/4	1/4	2/4	3/4	1/3	1/3	0/3	0/3	0/3	0/3	0/3	0/3	0/3	-
	6	0/3	0/3	0/3	2/3	3/3	1/3	0/3	0/3	0/3	0/3	0/3	0/3	0/3	-
	12 and older	0/3	0/3	0/3	0/3	0/3	0/3	0/3	0/3	0/3	0/3	0/3	0/3	0/3	-

Note: number of positive samples/number of samples tested.

**Table 4:** Localization and accumulation of PPRV in organs of infected animals (log_10_ TCID_50_/cm^3^).

Species (Age)	Sampling Time	GIT Mucosa *	Nasal Mucosa	Lungs	Spleen	Liver	Kidneys	Lymph Nodes **
Saiga (12–24 mo)	Day 3–5 illness	1.25–2.5	3.75	3.25	4.5	−	−	3.5–4.75
	1–3 h postmortem	≤1.0	1.5	1.75	2.75	−	−	0.75–2.5
Cameroon Goats (4–24 mo)	Day 3–5 illness	4.5–5.5	4.25	5.0	0.75	−	−	0.75–2.253
Local Goats (1–3 mo)	Day 3–5 illness	0.75–1.5	+	+	1.0	−	−	1.253
	1–3 h postmortem	−	+	−	−	−	−	+3
Local Goats (6–12 mo)	Day 3–5 illness	−	−	−	+	−	−	+3
Local Sheep (3–6 mo)	Day 3–5 illness	+	+	−	+	−	−	+3
Local Sheep (≥12 mo)	Day 3–5 illness	−	−	−	−	−	−	−

Note: * Gastrointestinal tract (abomasum, duodenum, jejunum, cecum, colon); ** Includes prescapular, suprascapular, mediastinal, and mesenteric lymph nodes; “+”—the virus was isolated at the 2–3 passage; “−”—the virus isolation result is negative.

**Table 5:** Virus shedding in animals infected with PPRV.

Animal Species and Age Group	Shedding Routes	Onset of Shedding	Duration of Shedding	Peak Virus Titer (lg TCID_50_/cm^3^)	Notes
Local goats (1–3 months)	Nasal, ocular, oral, feces	Days 1–2 post-fever	5–6 days (up to 8–9 in some)	0.75–2.5	Most shedding in acute/subacute cases; virus titers dependent on clinical severity
Local goats (6–12 months)	Nasal, ocular, feces	Days 2–3	2–3 days	Low titers; CPE in 2–3 passages	Shorter, weaker shedding; virus only isolated after blind passages
Local goats (≥12 months)	Feces	Days 2–3	2 days	Very low	Virus detected only in feces
Local sheep (1–3 months)	Nasal, ocular, oral, feces	Days 3–4	3–5 days	0.75–2.5	Similar to goat kids but shorter duration
Local sheep (6–12 months)	Nasal, ocular, saliva	Days 2–3	2–3 days	Very low	Virus not detected in feces
Local sheep (≥12 months)	Nasal only	Days 2–3	2–3 days	Trace amounts	Minimal shedding route
Cameroon goats (4–24 months)	Nasal, ocular, oral, feces	Day 2 post-fever	Until death (~6–9 days)	Nasal: 4.75; Ocular: 5.25; Oral: 3.75; Feces: 4.5	Consistently high titers in all secretions; major source of contamination
Saigas (6–24 months)	Nasal, ocular, oral, feces	Days 2–3 post-fever	4–7 days	Nasal: 4.0; Ocular: 4.25; Feces: 4.25	Virus titers increased toward the terminal stage
Recovered sheep/goats	None	–	0 days	Not detected	No virus carriage; confirmed by culture, PCR, and bioassay
All animals (general note)	Urine	–	–	Not detected	No virus detected in the urine of any animals

## Data Availability

The original contributions presented in this study are included in the article. Further inquiries can be directed to the corresponding authors.
